# Insights into Intrinsic Brain Networks based on Graph Theory and PET in right- compared to left-sided Temporal Lobe Epilepsy

**DOI:** 10.1038/srep28513

**Published:** 2016-06-28

**Authors:** Thomas Vanicek, Andreas Hahn, Tatjana Traub-Weidinger, Eva Hilger, Marie Spies, Wolfgang Wadsak, Rupert Lanzenberger, Ekaterina Pataraia, Susanne Asenbaum-Nan

**Affiliations:** 1 Department of Psychiatry and Psychotherapy, Medical University of Vienna, Austria; 2 Department of Biomedical Imaging and Image-guided Therapy, Division of Nuclear Medicine, Medical University of Vienna, Austria; 3 Department of Neurology, Medical University of Vienna, Austria; 4 Department of Neurology, General Hospital Amstetten, Austria

## Abstract

The human brain exhibits marked hemispheric differences, though it is not fully understood to what extent lateralization of the epileptic focus is relevant. Preoperative [^18^F]FDG-PET depicts lateralization of seizure focus in patients with temporal lobe epilepsy and reveals dysfunctional metabolic brain connectivity. The aim of the present study was to compare metabolic connectivity, inferred from inter-regional [^18^F]FDG PET uptake correlations, in right-sided (RTLE; n = 30) and left-sided TLE (LTLE; n = 32) with healthy controls (HC; n = 31) using graph theory based network analysis. Comparing LTLE and RTLE and patient groups separately to HC, we observed higher lobar connectivity weights in RTLE compared to LTLE for connections of the temporal and the parietal lobe of the contralateral hemisphere (CH). Moreover, especially in RTLE compared to LTLE higher local efficiency were found in the temporal cortices and other brain regions of the CH. The results of this investigation implicate altered metabolic networks in patients with TLE specific to the lateralization of seizure focus, and describe compensatory mechanisms especially in the CH of patients with RTLE. We propose that graph theoretical analysis of metabolic connectivity using [^18^F]FDG-PET offers an important additional modality to explore brain networks.

Temporal lobe epilepsy (TLE) is a common neurological disorder, which is defined by characteristic clinical features and a typical seizure semiology. Associated morphological brain alterations may be caused by recurrent seizure propagation and deafferation of brain regions connected to the hippocampus^1^,^2^. Structural and functional magnetic resonance imaging (sMRI, fMRI) investigations have revealed widespread gray and white matter alterations in patients with TLE within temporal as well as several extra-temporal cortical and subcortical brain regions^3^,^4^. Studies exploring brain connectivity have revealed profound changes in patients with TLE. Findings implicate altered interaction of brain networks, which is provoked through seizure spread and generated by the epileptic onset zone in the hippocampus^5^,^6^. Therefore, based on numerous neuroimaging studies, TLE has been classified as a disorder of disturbed neural networks.

Network analyses in TLE have been performed by determination of structural or functional connectivity using interregional correlation analysis. Cortical thickness analysis has been used to model structural connectivity, investigating connectivity patterns and correlations between brain areas, respectively in healthy control subjects and in different neuropsychiatrical disorders^7^. Functional networks were investigated using fMRI^5^,^8^,^9^,^10^ or electroencephalography (EEG)^7^,^11^. It has been demonstrated that connectivity abnormalities in TLE are not restricted to the pathological hippocampus, as changes were found in ipsi- and contralateral brain regions, which supports the network theory of TLE^12^.

However, only a few studies utilizing nuclear medicine methodology to explore connectivity on a network level have been published. Studies applying positron emission tomography (PET) focus mainly on metabolic connectivity using fluorine-18 [^18^F] fluorodeoxyglucose (FDG) ([^18^F]FDG PET). [^18^F]FDG PET is one of the most accurate imaging methods to evaluate the functional deficit zone^13^, in other words, to detect a brain region with the most distinct glucose hypometabolism. So far the underlying mechanisms of this hypometabolism in the seizure focus region have not been identified. However, neuronal loss, diaschisis and decreased blood-brain barrier glucose transporter activity have been proposed to underlie differences in FDG uptake in patients with TLE^14^,^15^,^16^,^17^. Interregional correlation analyses of glucose metabolism rates have been used to explore brain connectivity in healthy control subjects, Alzheimer’s disease, Down’s syndrome, autism and obsessive-compulsive disorder as well as in deafs and patients with TLE^18^,^19^,^20^,^21^,^22^,^23^,^24^,^25^, which suggests that connectivity can be assessed by FDG uptake and that metabolic connectivity and therefore large scale brain networks are altered in neuropsychiatric disorders. The underlying hypothesis is that brain regions with significant correlated glucose turnover, measured with [^18^F]FDG PET, are metabolically connected.

Structural and functional whole brain connectivity in patients with TLE have been explored using different neuroimaging modalities and graph theory analysis. Morphological MRI parameters such as cortical thickness or grey matter volume as well as white matter volume have been used to examine structural connectivity^26^,^27^ and fMRI or EEG to delineate functional connectivity^6^,^28^.

Due to distinct hemispheric organization, morphological and functional differences have been observed in left-sided TLE (LTLE) and right-sided TLE (RTLE)^29^,^30^,^31^. If TLE is assumed to be a network disorder, then the question arises whether patients with RTLE and right-sided hippocampal sclerosis demonstrate different alterations of the connectome than patients with a left-sided one. Differences between patients with left- and right-sided hippocampal sclerosis are indicated by clinical symptomatology as well as in several imaging studies^7^,^8^,^31^. Patients with LTLE demonstrate more severe deficits in verbal memory and language processing; patients with RTLE exhibit deficits in non-verbal, visual memory^32^. Former MRI investigations already indicated differences between these two types of TLE using structural or functional connectivity measurements^33^,^34^.

To the best of our knowledge, this is the first study to investigate metabolic connectivity in patients with RTLE and LTLE based on glucose metabolism and using a graph theoretical approach to compare network properties of two faces of an apparently lateralized disease. We expected to gain new insight into hemispheric properties which might help to understand the manifold clinical image of TLE and hypothesize that specialization of right and left hemisphere (as spatial orientation and speech) includes different projections and networks.

## Results

### Clinical data

Patients with RTLE did not differ significantly from LTLE in sex, age at PET and age at operation (Table 1). Further patient groups did not show significant differences in age at onset, duration of disease, seizure frequency and surgery outcome (ranks were set according to ILAE classification 2001^35^). We found significant differences in age between both patient groups and HC (Table 1). Outcome data of two patients with RTLE and two patients with LTLE are missing due to non-appearance at follow up visits, or follow up-time.

### Glucose metabolism

**Figure 1:**
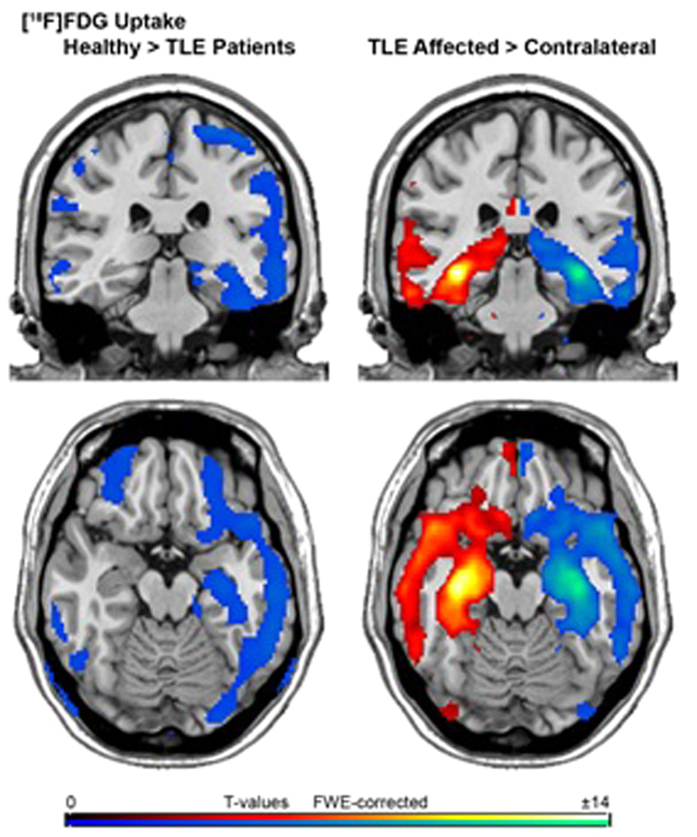
A coronal and transversal view of differences in FDG uptake between patients with TLE (patients with RTLE and LTLE pooled as one group) and the healthy control group (images on the left side) and between the affected and the contralateral hemisphere in all patients (RTLE and LTLE pooled together, images on the right side). We found most pronounced decrease of FDG uptake in the temporal lobe including hippocampus of the affected hemisphere (AH) and otherwise spread throughout the entire cortex, except for the ant/middle cingulate cortex, in the AH and contralateral hemisphere (CH), when comparing patients with TLE to healthy controls. Furthermore, when comparing AH to CH in all patients pooled together, we detected a significant decrease in FDG uptake in the AH, including hippocampus, parahippocampus, inferior, medial and superior temporale lobe, temporal pole, fusiform gyrus, amygdala and the insula as well as thalamic, caudatus, anterior cingulate cortex, inferior orbitofrontal cortex and precuneus/posterior cingulum. The colour table shows the corresponding t-value (t > 5.7, P < 0.05; FWE-corrected at cluster level).

Comparing patients with TLE (RTLE and LTLE pooled together as one group) to healthy controls (HC), we found most pronounced decrease in FDG uptake in the temporal lobe including hippocampus in the affected hemisphere (AH) and otherwise spread throughout the entire cortex, except for the anterior and middle cingulate cortex, in the AH and the contralateral hemisphere (CH) (P < 0.05 FWE-corrected cluster level, corrected for age and sex; Fig. 1).

Furthermore, when comparing AH to CH in all patients (RTLE and LTLE pooled together), we found a significant decrease in FDG uptake in the hemisphere with the seizure focus. Brain regions with decreased FDG uptake included the hippocampus, parahippocampus, inferior, middle and superior temporale lobe, temporal pole, fusiform gyrus, amygdala and the insula as well as thalamic, caudatus, anterior cingulate cortex, inferior orbitofrontal cortex and precuneus/posterior cingulum (P < 0.05 FWE-corrected cluster level; Fig. 1).

### Graph theoretical properties

Globally, the type of network did not discriminate RTLE from LTLE and patients with TLE of both groups from HC, as groups displayed similar small worldness properties almost across the entire range of sparsities.

**Figure 2:**
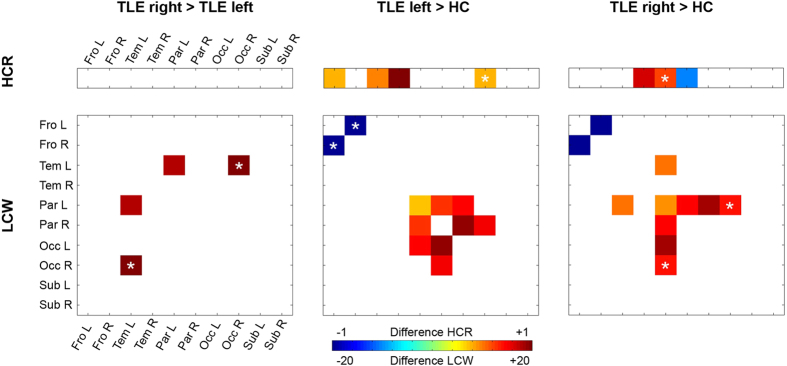
Graph theroretical properties, hemispheric connectivity ratio (HCR) on the upper hand and lobar connectivity weight (LCW) on the lower hand, are depicted for all group comparisons; from left to right: TLE right > TLE left, TLE left > HC, TLE right > HC. The HCR was analyzed as the ratio between intra- and interhemispheric connections for a particular lobe, whereas a higher ratio states increased connectivity of a lobe within the hemisphere. The LCW was calculated to quantify connections between any two given lobes. The colour table shows differences in HCR and LCW; blue indicates lowest and red highest molecular connectivity. Asterix in the coloured square depicts a trend towards differences in HCR and LCW for covariates of sex or age.

Reflecting the relationship between lobes within and across hemispheres, the hemispheric connectivity ratio (HCR) analysis revealed no significant differences when comparing patients with RTLE to LTLE. We observed higher HCR in the temporal lobe in the AH in both patient groups compared to HC and in addition in the CH in LTLE. We saw significant higher HCR in the frontal lobe of the CH and in the occipital lobe of the AH just in patients with LTLE (trend-wise after correction for sex in the occipital lobe). Further results demonstrated significant lower HCR in the parietal lobe of the AH in patients with RTLE compared to HC and a trend towards higher HCR in the parietal lobe of the CH (Fig. 2).

**Figure 3:**
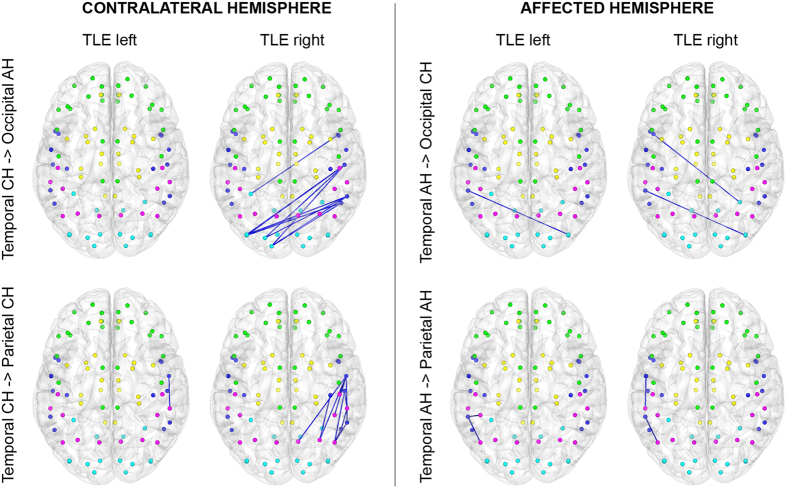
Lobar connectivity weights (LCW) in patients with RTLE and LTLE. Significantly stronger connections were found in patients with RTLE between the temporal and the parietal lobe of the contralateral hemisphere and a trend towards higher LCW in the contralateral temporal with the occipital lobe of affected hemispheres (left panel). In contrast, no difference was observed for connections of the affected temporal lobe between the two patient groups. The left hemisphere represents the contralateral hemisphere for both groups shown in radiological convention (right hemisphere in all images is the contralateral hemisphere). Different color dots indicate different lobes (green = frontal, dark blue = temporal, yellow = subcortical/limbic, pink = parietal, light blue = occipital), whereas specific dots depict AAL (automated anatomical labelling)-based atlas^56^ brain regions.

On finer levels of granularity, however, group differences were predominantly observed in the CH rather than in the affected one. We found significantly higher lobar connectivity weight (LCW) in RTLE compared to LTLE for connections between the temporal and parietal lobe of the CH and a trend for higher LCW of the contralateral temporal lobe with the occipital lobe of the AH (Figs 2 and 3). Significantly higher LCWs were observed in both patient groups compared to HC in the parietal lobe in the CH with the occipital lobe of both hemispheres (LTLE and HC only in the CH and trend-wise results after correction for age in the parietal lobe of the CH to the occipital lobe of the AH in patients with RTLE) as well as in patients with LTLE in the parietal lobe in both hemispheres with the occipital lobe of the CH. When comparing RTLE with HC, we were able to demonstrate significant higher LCWs connections of the temporal to the parietal lobe in the CH. Lower LCW was found between the frontal lobes of both hemispheres (trend-wise after correction for sex in in patients with LTLE compared to HC).

**Figure 4:**
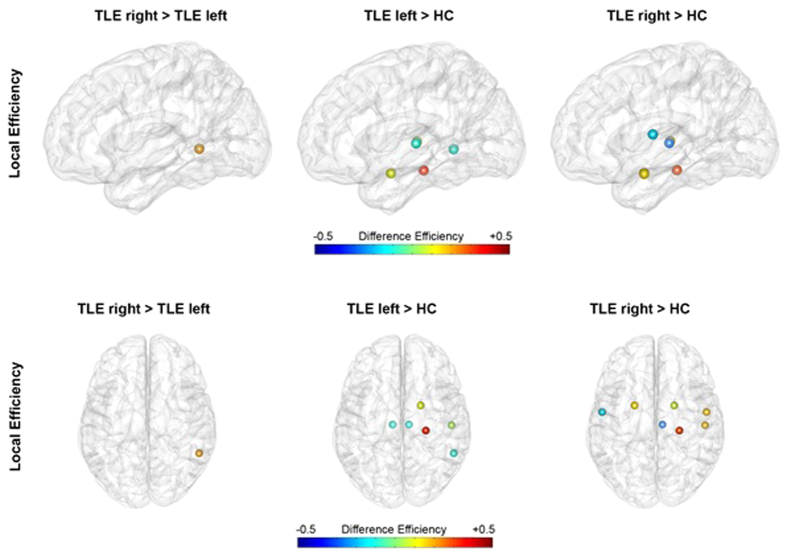
Local graph theory parameters showing significant differences between patients with RTLE and LTLE and between the two patient groups and the healthy control group. Upper images depict the brain from the left side, lower images from above. The left hemisphere represents the contralateral hemisphere for both groups shown in radiological convention (right hemisphere in all images is the contralateral hemisphere). Local efficiency was found to be higher in the middle temporal region of the contralateral hemisphere in patients with RTLE, when comparing patients groups. Compared to HC, both patient groups showed alteration of local efficiency in several brain regions, predominantly in the contralateral hemisphere.

On a regional level, these findings were accompanied with higher local efficiency in the middle temporal cortex of the CH in patients with RTLE, when comparing patients groups. Results further showed altered local efficiency in patients with LTLE compared to HC predominantly in the CH, more specific in the temporal lobe (trend-wise after correction for sex in the middle temporal lobe) and in the amygdala of the CH, and in the thalamus of both hemispheres. Pronounced differences in local efficiency of the CH in patients with RTLE compared to HC, including the temporal and parietal lobe and the thalamus, as well as the amygdala and the operculum rolandic of both hemispheres were delineated (Fig. 4).

After correction for duration of epilepsy and seizure quantity, we observed similar results when comparing patient groups. Only after correcting for duration of epilepsy, LCWs changed from a trend towards significant differences between patients with RTLE compared to LTE for connections between the temporal lobe of the CH to the occipital lobe of the AH.

## Discussion

The aim of the present neuroimaging study was to investigate metabolic brain networks in patients with RTLE and LTLE using graph-theoretical analyses. To differentiate specific metabolic connectivity inherent to lateralization of epileptic focus, we compared glucose metabolism throughout the affected and contralateral hemispheres, utilizing metabolic imaging and graph theoretical analyses in TLE. While some of our findings underline established hypothesis regarding the pathophysiologic changes of TLE, others reveal novel insights into reorganizational processes within the CH and lobar connectivity in TLE.

Except for similar small worldness, connectome findings demonstrated differences between patient groups in the affected and contralateral hemisphere in HCR and LCW properties. More specifically, we found more wide spread differences in intra- and interhemispheric molecular connectivity in patients LTLE than in RTLE, including lobes in the AH and the CH. Results further showed higher lobar molecular connectivity in the CH in patients with RTLE than in LTLE and similar lobar molecular connectivity differences when comparing each patient group to HC. On a nodal level, patients with RTLE and LTLE displayed altered local efficiency in the CH. Taken together, when comparing the patient groups, findings demonstrated a more pronounced connectivity in the CH in patients with RTLE, substantiating the notion of distinct neuronal activity related to side of seizure focus.

[^18^F]FDG PET neuroimaging studies investigate brain activity through observation of glucose metabolism at the synaptic level, which is the functional neuronal component of highest glucose turnover^20^. [^18^F]FDG PET is used in epilepsy centers to define seizure focus in temporal lobe epilepsy, and beyond that, to display dysfunctional metabolism throughout the brain in epilepsy. Over the last three decades neuroimaging studies have demonstrated metabolic connectivity based on interregional correlation analyses in healthy control subjects and in various neuropsychiatric disorders^20^,^24^,^36^, driven by the hypothesis that brain regions are metabolically coupled when glucose uptake correlates between different brain regions. Furthermore, it has been demonstrated that local brain activation is highly correlated with functional connectivity in healthy controls, measured with fMRI and EEG^37^, and that the correlation between glucose metabolism and functional connectivity may be abnormal in patients with TLE^25^. Graph theoretical investigations in a pilocarpine-induced epilepsy animal model revealed irregularities in global metabolic connectivity, providing more specific information on neural activity in TLE^38^.

Imaging studies investigating networks using graph theoretical approaches exhibited preserved small world topology in patients with TLE, hence, maintaining the ability to incorporate both segregated and integrated information^39^,^40^. To minimize costs while retaining efficiency, the human brain incorporates information among distant regions via short-cut nodes, or so-called hubs, and highly specialized brain regions^41^. In line with previous evidence^40^, we found similar small-world topology across all investigated subjects.

Regardless of lateralization, altered connectivity in the hemisphere opposing the epileptic zone was frequently seen in patients with TLE^42^,^43^. Graph investigations mainly observed attenuated global efficiency in TLE. It is conceived that in order to prevail whole brain functional connectivity and compensate for neuronal damage, local connectivity was repeatedly found to be enhanced in the affected as well as in the CH^8^,^42^,^43^. This suggests TLE as a network disorder, which projects synchronous discharges ubiquitously throughout the brain. Subsequently this accounts for the dissemination of damage to neurons within the brain and to a loss of connectivity^1^,^2^. The increased wiring and re-routing in brain regions of CH is interpreted as the ability of the nervous system to modify both structure and function over time in response to seizure-induced neuronal damage. Our finding therefore emphasizes compensatory processes engaged in TLE.

In line with previous studies^4^,^8^,^40^, the results showed significant differences in lobar molecular connectivity between patient groups and in hemispheric molecular connectivity in patients with LTLE and RTLE compared to HC. Analyses of hemispheric connectivity demonstrated higher intra- than inter-hemispheric connectivity in the temporal and the frontal lobe of the CH as well as in the temporal and the occipital lobe of the AH between patients with LTLE and HC. We found higher HCR in the temporal lobe and lower HCR in the parietal lobe of the AH as well as a trend towards higher HCR in the parietal lobe of the CH in patients with RTLE in relation to HC. Patients with TLE of both groups demonstrated higher molecular connectivity generated in the temporal lobe of the AH, when compared to HC. Focusing on LCW, we saw higher lobar molecular connectivity between the temporal and parietal lobes of the CH in patients with RTLE and higher LCW in the parietal and the occipital lobe in both patient groups compared to HC. In the frontal lobe, we observed lower lobar molecular connectivity between the frontal lobes of the affected and contralateral hemisphere in LTLE and RTLE compared to HC, which is in line with fMRI findings that show an interhemispheric connectivity in the frontal lobe^33^,^44^. The predominantly observed differences exhibited in hemispheric and lobar connectivity strengthen established findings that describe a partial distinct neuronal signature for RTLE and LTLE.

Brain morphological changes are generally altered in patients with TLE, showing tendencies towards a more evident and widespread impairment in patients with LTLE than in those with RTLE^4^,^45^,^46^. Compared to healthy controls, patients with TLE displayed decreased functional connectivity in the AH and increased functional connectivity in CH^42^,^43^. We observed disrupted functional connectivity more pronounced in LTLE compared to RTLE, while connectivity was found to be increased in the unaffected hippocampus in patients with RTLE. Wang and colleagues^43^ detected significant differences in nodal parameters in the affected and contralateral hemisphere in patients with RTLE and LTLE. In a recent fMRI study comparing lateralized TLE, reductions in limbic network connectivity were shown in both RTLE and LTLE patients, though elevated connectivity was demonstrated in the CH. In addition, local efficiency and clustering coefficient were reduced in the right hippocampus in patients with LTLE and increased betweenness centrality in the left hippocampus in patients with RTLE^31^. Our results are therefore in line with literature, suggesting more pronounced compensatory mechanisms in the CH in patients with RTLE and in the AH in patients with LTLE.

On a local network level, we detected significant differences in local efficiency, which is a nodal parameter that allows quantifying the importance of nodes for the interaction within the network. In comparison to LTLE, patients with RTLE demonstrated elevated molecular connectivity within the temporal lobe of the CH, more specific in the middle temporal region. Compared to HC, we found alterations predominantly in the CH for both patient groups, comprising regions within the temporal lobe, the amygdala and the thalamus in both hemispheres. Elevated network properties in the CH are hypothesized to compensate for damaged structure and impaired networks in the AH, presenting compensational mechanism. Several investigations associate cognitive deficits with imaging findings in patients with TLE, linking more profound pathophysiological changes in patients with left lateralized seizure focus to greater cognitive impairment in this patient group^47^,^48^. Thus, higher local efficiency and LCW in the CH in patients with RTLE compared to HC may present a higher potential for the development of compensational mechanisms in this patient group.

A [^18^F]FDG PET study demonstrated the ability to discriminate lateralization of seizure focus and to predict seizure prognosis via analysis of regional and global brain metabolism in patients with TLE^49^. We first compared patients with TLE of both groups to HC. We observed a reduced FDG uptake in patients with TLE, including most pronounced the hippocampus of the focus side as well as in the entire cortex, except for the anterior and middle cingulate cortex. Comparing FDG uptake between the AH to the CH of both patient groups, our results demonstrated reduced metabolism in the affected temporal lobe, which is in line with previous studies^13^. These structures include included the hippocampus, parahippocampus, inferior, medial and superior temporale lobe, temporal pole, fusiform gyrus, amygdala and the insula as well as thalamic, caudatus, anterior cingulate cortex, inferior orbitofrontal cortex and precuneus/posterior cingulum. This suggests disrupted regional and global neuronal networks in epilepsy as a cause for seizure propagation^50^.

Our results confirm the feasibility of [^18^F]FDG PET for the analyses of metabolic connectivity using graph theory. A limitation of this study is an unknown effect of ongoing antiepileptic therapy or of subclinical seizure activity on network parameters. Present therapy was not evaluated separately, but should be equalized statistically for both patient groups. Further, seizures during PET investigations were excluded by observation, but there was no online EEG during PET acquisition. Consequently sleep or drowsiness during PET could not be excluded as well. Finally, flipping of the hemisphere in one patient group was required for analysis reasons, i.e., to have the affected hemisphere on the same side for all patients. Hence, one limitation is that we cannot rule out that our results are still influenced by inherent structural and/or functional differences between left and right hemispheres.

The results of this investigation suggest significant altered metabolic networks in patients with TLE, which supports MRI investigations and captures similar information through utilizing [^18^F]FDG PET and graph theory. The results of this graph theoretical approach revealed a distinct neuronal pattern, dependent on lateralization of epileptogenic focus. Differences in hemispheric and lobar molecular connectivity have been observed between patients and HC and between RTLE and LTLE in the affected and contralateral hemisphere, confined to the temporal, parietal, frontal and occipital lobe. Higher contralateral molecular connectivity is found more pronounced in patients with RTLE, implicating more compensatory mechanisms opposing to the damaged neuronal tissue subsequent to seizures in patients with RTLE compared to LTLE. Hence, the results of this neuroimaging study underline the feasibility of [^18^F]FDG PET data for measurement of connectivity across brain regions and for further investigation of the pathophysiological mechanisms in TLE.

## Materials and Methods

### Subjects

62 drug resistant patients with unilateral TLE who underwent [^18^F]FDG PET for pre-surgical assessment between 1999 and 2009 were recruited from the Epilepsy Monitoring Unit of the Department of Neurology, Medical University of Vienna. The diagnosis was based on clinical history, physical and neurological exams, results of prolonged video-EEG monitoring including clinical seizure semiology and interictal and ictal EEG results, high resolution MRI and inter-ictal [^18^F]FDG PET. 30 patients had RTLE (mean age at PET acquisition in years ± SD = 36.90 ± 9.50; female/male = 14/16) and 32 patients had LTLE (40.50 years ± 11.25; 14/18). Except for TLE, patients had no history of any other neurological disorder. Subsequently to the comprehensive diagnostic procedure, all patients underwent selective amygdalo-hippocampectomy, except for two patients with RTLE, who underwent anteriormedial temporal resection, and hippocampal sclerosis, indicated in MRI, was confirmed histologically in all patients. Demographic patient data was assessed from patients’ clinical work-up (see Table 1). For comparison, 31 healthy controls were pooled from previous PET studies (27.33 years ± 6.43; 11/20), without any neuropsychiatric or somatic disorders.

We determined duration of disease as age at PET minus age at first seizure occurence, seizure frequency according to Aull-Watschinge *et al*.^51^, distinguishing between more than one seizure per week (high seizure frequency) and less than to one seizure per week (low seizure frequency), and outcome according to ILAE classification 2001^35^, distinguishing between outcome classification 1 (=0) and outcome classification 2–6 (=1).

All experimental protocols were approved by the ethical board of the Medical University of Vienna, Vienna, Austria, and were performed in accordance with approved guidelines and regulations. Written informed consent was obtained by all participants.

### [^18^F]FDG PET

[^18^F]FDG was prepared using a fully automated platform (FASTlab^®^, GE Healthcare) according to Hamacher *et al*.^52^. PET scans were performed using a GE Advance PET Scanner with a spatial resolution of 4.36 mm full-width at half-maximum 1 cm next to the center of the field of view (matrix = 128 × 128, 35 slices, voxel size = 3.125 × 3.125 × 4.25 mm). FDG was injected intravenously at a mean dose of 3.54 ± 0.86 MBq/kg body weight. For attenuation correction a 5 min transmission scan was carried out with retractable ^68^Ge rod sources. The emission measurement was done in static 3D mode, which started 30 min after injection and lasted 10 minutes. Imaging procedures were carried out under resting and awake conditions and carefully controlled for ictal events. To minimize head movements, patient’s head was placed in a polyurethane head mould and straps were placed around the chin and the forehead.

### Imaging Analysis

PET images were spatially normalized to a tracer-specific template in MNI-space using SPM8 (http://www.fil.ion.ucl.ac.uk/spm/). Images were normalized to standard uptake values by dividing each voxel’s activity by the injected dose per kg body weight. For network analyses, images were further normalized to the average brain uptake^53^,^54^,^55^. PET images of the RTLE had to be left-right flipped for further data analysis, to ensure a homogeneous group with all patients having the seizure focus on the same side. Afterwards, normalized glucose uptake values were extracted for 88 regions of interest using an AAL (automated anatomical labelling)-based atlas^56^. Correlation matrices were constructed by calculating Pearson’s correlation coefficient between every region pair across for each group (RTLE, LTLE and HC separately), which served as basis for the subsequent graph theoretical analyses^57^. To rule out that the observed effects were potentially driven by nuisance variables, we included illness duration and seizure frequency as well as age at PET and sex into the construction of connectivity matrices by using partial correlations in a subsequent analysis. Since the number of patients with a negative outcome after surgery is rather small (RTLE = 4, LTLE = 7), this variable was not included in the evaluation due to insufficient statistical power.

### Graph Theory

Compound network analyses are essential to explore relationships of connected brain regions. An abundance of connectivity measures have emerged over the past years to describe anatomical and functional connectivity on both global and local levels. In this investigation we selected several network properties, describing the network modalities in TLE and HC at global, hemispheric, lobar and local levels. Network measures as small-worldness, HCR, LCW and local efficiency^39^,^58^ are all implicated to be relevant to the underlying mechanisms of unilateral TLE. Together, these measures comprehensively describe network connectivity at different levels of granularity (i.e., global, hemispheric, lobar and regional levels). Small-worldness represents a network with high global and local efficiency, hence, exhibiting higher clustering and similar characteristic path length as compared to random networks. Here, we used 100 Erdös-Renyi random networks with the same number of nodes and edges. To assess networks at hemispheric and lobar levels, ROIs were assigned to frontal, temporal, parietal, occipital or subcortical/limbic lobes for the left and right hemisphere. The HCR was then calculated as the ratio between intra- and interhemispheric connections for a particular lobe, with a higher ratio standing for an increased connectivity of the lobe within the hemisphere. To quantify the connections between any two given lobes, we computed the LCW. This is given as the sum of connections within (e.g., frontal left–frontal left) or between different lobes (e.g., frontal left–frontal right, frontal left–temporal left). Finally, local efficiency served for an evaluation at the regional level. These represent the connections between neighbours of a node, and local information transfer of a region, respectively. All graph metrics were computed for binarized networks at densities of 10–50% in 5%-steps using the brain connectivity toolbox (28) and Matlab R2011a (MathWorks Inc., Natick, MA, USA). Brain networks were visualized with the Brain Net Viewer^59^.

### Statistical Analysis

Whole brain statistics were computed in SPM8 (P < 0.05, FWE corrected for multiple comparisons at cluster level following p < 0.001 uncorrected voxel level). First, clinical and epidemiological data were compared with unpaired two-tailed t-tests and Mann-Whitney U Tests. Differences in metabolism between affected and contralateral hemisphere were assessed by paired t-tests in the pooled patient group using SPM8. Graph metrics characterizing metabolic connectivity networks were evaluated using N = 5000 random permutations^57^. To assess comparisons between patient with RTLE and LTLE as well as between both patient groups and HC, the graph metrics were first computed for the true group memberships (observed effect). Afterwards, patients were randomly assigned to one of the groups within each permutation and network metrics were computed again (permuted effect). Network metrics should be considered significant if changes are observed across a wide range of network densities. This was realized by averaging network metrics across all sparsities (after z-transformation to ensure the same scale across network densities). Next, a single-threshold test was applied which accounts for the multiple comparison problem^58^,^60^,^61^. The maximum of the network metric across all tests (e.g., brain regions, lobes, etc) was obtained for the true group membership and each permutation. Using this distribution of the maximum effect, the significance threshold at α = 0.05 and the trend threshold at α = 0.1 were computed as the 95th percentile and the null hypothesis was rejected for any test (e.g., brain region) exceeding this threshold. This procedure has been shown to have strong control over type I errors, resulting in FWE-corrected p-values^60^,^61^. Since the control group was pooled from different previous studies and where thus not explicitly matched to patients, all comparisons of glucose metabolism and graph theoretical properties between patients with TLE and HC were corrected for sex and age.

## Additional Information

**How to cite this article**: Vanicek, T. *et al*. Insights into Intrinsic Brain Networks based on Graph Theory and PET in right- compared to left-sided Temporal Lobe Epilepsy. *Sci. Rep.* **6**, 28513; doi: 10.1038/srep28513 (2016).

## Figures and Tables

**Table 1:** Demographic data of included patients with mesial temporal lobe epilepsy.

	RTLE	LTLE	HC
f/m	14/16	14/18	11/20
age at PET (y, mean ± SD)	36.90 ± 9.50	40.50 ± 11.25	27.33 ± 6.43^*#^
age at OP (y, mean ± SD)	37.90 ± 9.60	41.23 ± 11.36	–
follow up (y, mean ± SD)	5.62 ± 1.94	6.22 ± 3.20	–
duration (y, mean ± SD)	25. 60 ± 14.21	26.20 ± 14.01	–
age of onset (y, mean ± SD)	11.61 ± 7.91	14.31 ± 10.77	–
outcome (median)	1	1	–
seizure (median)	1	1	–

Legend: RTLE = right-sided mesial temporal lobe epilepsy, LTLE = left-sided mesial temporal lobe epilepsy, f = female, m = male, SD = standard deviation, y = years, OP = operation. Outcome is classified to ILAE classifications (1 outcome = 0, 2–6 outcome = 1). Seizure frequency is classified to high seizure frequency (=1) and low seizure frequency (=0). *p < 0.05 for HC vs RTLE, ^#^p < 0.05 for HC vs LTLE using Mann-Whitney U-test and unpaired two-tailed t-test when appropriate. RTLE and LTLE did not differ significantly in any of the above variables.
